# Stimulated Hyperinsulinemia Is Independently Associated with Higher Serum DHEAS in PCOS: A Retrospective Study

**DOI:** 10.3390/jcm14176246

**Published:** 2025-09-04

**Authors:** Nicoleta Baculescu, Serban Radian, Dana Manda, Cristina Georgiana Serban, Dan Alexandru Niculescu, Monica Livia Gheorghiu, Florin Grigorescu, Catalina Poiana

**Affiliations:** 1 Department of Endocrinology, Carol Davila University of Medicine and Pharmacy, 020021 Bucharest, Romania; serban.radian@umfcd.ro (S.R.); cristinageorgianaserban1996@gmail.com (C.G.S.); dan.niculescu@umfcd.ro (D.A.N.); monica.gheorghiu@umfcd.ro (M.L.G.); endoparhon@gmail.com (C.P.); 2 First Endocrinology Department, C.I. Parhon National Institute of Endocrinology, 011863 Bucharest, Romania; 3 Molecular Cellular and Structural Endocrinology Laboratory, C.I. Parhon National Institute of Endocrinology, 011863 Bucharest, Romania; dana.manda@gmail.com; 4 Institut Convergences Migrations, Collège de France, 93322 Paris-Aubervilliers, France; florin.grigorescu@inserm.fr

**Keywords:** DHEAS, adrenal hyperandrogenism, hyperinsulinemia, PCOS

## Abstract

**Background/Objectives**: Increased dehydroepiandrosterone sulfate (DHEAS) is used as a diagnostic marker of hyperandrogenism in women with polycystic ovary syndrome (PCOS). The mechanisms of adrenal hyperandrogenism in PCOS include hyperinsulinism as a potential stimulator, but results of studies associating insulinemia with DHEAS in PCOS are conflicting. The objective of this study was to evaluate the factors associated with DHEAS levels in PCOS, focusing on insulinemia. **Methods**: We performed a cross-sectional retrospective study in a total of 257 patients with PCOS (Rotterdam criteria) evaluated in our tertiary center of endocrinology. Clinical and biochemical parameters included body mass index (BMI), serum DHEAS, total testosterone and sex hormone-binding globulin (SHBG), insulin and glycaemia at fasting and 2 h during the oral glucose tolerance test (OGTT), and homeostasis model assessment for insulin resistance (HOMA-IR) was calculated. **Results**: The comparative analysis of PCOS divided into DHEAS tertiles revealed that patients in the upper tertile were younger (*p* < 0.05) and had higher 2 h insulin in the OGTT (*p* < 0.05) than the lower tertile, while fasting insulin and HOMA-IR were not different. DHEAS correlated negatively with age (r = −0.146, *p* < 0.05) and positively with 2 h insulinemia (r = 0.246, *p* < 0.001), while fasting insulin and HOMA-IR did not correlate with DHEAS in all PCOS. In stepwise linear regression models, 2 h insulin remained a positive independent predictor for DHEAS only in non-obese PCOS (*p* < 0.0001). **Conclusions**: Our data indicate a positive association between stimulated insulin and DHEAS in PCOS. Two-hour insulin in OGTT was an independent predictor of DHEAS in non-obese PCOS, suggesting that DHEAS might be a reliable marker for the stimulatory insulin effect on adrenal steroidogenesis in non-obese PCOS patients.

## 1. Introduction

Polycystic ovary syndrome (PCOS) is the leading cause of reproductive and metabolic dysfunction in young women, affecting up to 15% of the female population of reproductive age worldwide, in function of the diagnostic criteria [1]. Hyperandrogenism is a key feature of the disorder; however, insulin resistance and hyperinsulinemia are also hallmarks of PCOS. Although the vast majority of studies were focused on the mechanisms of ovarian hyperandrogenism in this syndrome, it is now certain that adrenal androgen secretion is also enhanced in PCOS [2,3,4]. The adrenal contribution to circulating androgen excess was variable in different PCOS studies [5]; however, in a more recent analysis, adrenal androgens were the dominating serum androgens in women with PCOS and significantly elevated than in healthy controls [6]. Therefore, identifying the driving factors of adrenal hyperandrogenism in PCOS gains an important role in understanding the pathogenic mechanisms of this syndrome.

While the inherited defects of adrenal steroidogenic enzymes were excluded [7,8], an increasing number of studies indicate that elevated adrenal androgen secretion in PCOS is not related to altered pituitary responsivity to corticotropin-releasing hormone (CRH) or to increased sensitivity of adrenal androgens to adrenocorticotropic hormone (ACTH) stimulation [9,10]. Adrenal hyperandrogenism could arise from intrinsic activation by intra-adrenal factors, or it could be secondary to peripheral influences. In this context, several studies evaluated the effect of insulin on adrenal androgen secretion in PCOS, but results remained conflicting in both cross-sectional [11,12,13,14] and experimental studies of short-term induced hyperinsulinemia [15,16,17,18,19,20,21,22].

Dehydroepiandrosterone sulfate (DHEAS) is considered a specific diagnostic marker of adrenal hyperandrogenism because the sulfotransferase converting DHEA to DHEAS is specifically expressed in the adrenal cortex and not in the ovary [23]. In addition, high production rate and low metabolic clearance determining small diurnal fluctuations in blood levels, together with easy and accurate measurement by direct commercial immunoassays, make DHEAS a reliable marker of adrenal hyperandrogenism [10,24].

Therefore, we used DHEAS as a marker of adrenal hyperandrogenism and performed a cross-sectional analysis in a PCOS population from Romania, focusing on the association between insulin and DHEAS levels.

## 2. Subjects and Methods

**Figure 1:**
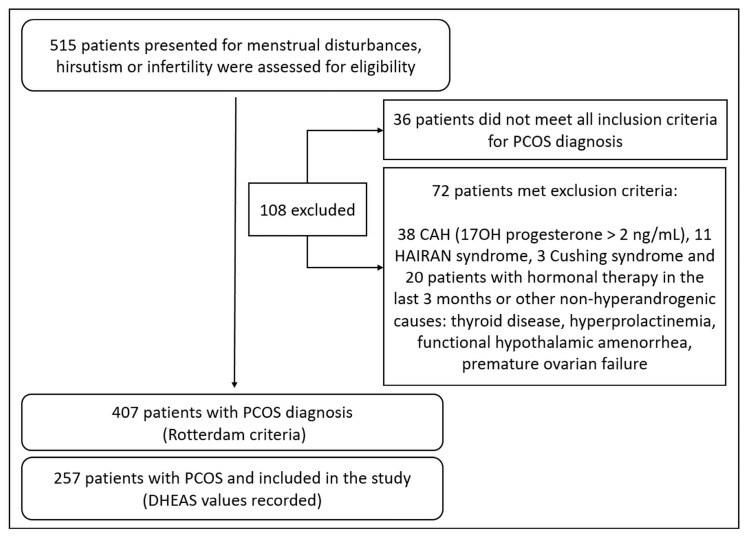
Flow diagram representing the selection of PCOS subjects for the study.

A total of 257 Romanian PCOS patients aged 16–41 years were analyzed retrospectively. These patients were selected from our database of PCOS patients evaluated at the “C.I. Parhon” National Institute of Endocrinology, Bucharest, between 2011 and 2020. The flow diagram of potentially eligible patients and criteria of exclusion is shown in Figure 1. A total of 515 subjects were evaluated because of menstrual disturbances, hirsutism or infertility, and PCOS was diagnosed in 407 of them. For the purpose of this study, we selected patients with DHEAS measurements.

The diagnosis of PCOS was established according to the Rotterdam 2003 consensus [25]. Oligo-anovulation was indicated by the presence of oligomenorrhea (i.e., less than eight menstrual bleeding events during the last year) or amenorrhea (i.e., no menstrual bleeding during the last 3 months). Biochemical hyperandrogenism was defined as total testosterone (TT) ≥ 0.75 ng/mL, free-androgen index (FAI) ≥ 8 and/or DHEAS higher than age-specific upper normal limits of our laboratory (i.e., DHEAS ≥ 321 µg/dL for the patients aged 18–21 yr, DHEAS ≥ 391 µg/dL for the patients aged 21–30 yr and DHEAS ≥ 266 µg/dL for the patients older than 30 yr). Clinical hyperandrogenism was diagnosed according to the presence of hirsutism at a modified Ferriman–Gallwey (mFG) score of 8 or higher [26]. Polycystic ovarian morphology was defined when at least one ovary had 12 or more follicles measuring 2–9 mm in diameter or a volume of greater than 10 cm^3^ (milliliters) [25]. Ovarian morphology and size were determined by transvaginal ultrasound or, rarely, by transabdominal ultrasound, performed on the same days as clinical and biochemical evaluation. Exclusion criteria for the study were active thyroid disease, hyperprolactinemia, functional hypothalamic amenorrhea, premature ovarian failure, Cushing’s syndrome, late-onset adrenal congenital hyperplasia due to 21-hydroxylase deficiency [i.e., 17α-OH-progesterone levels > 2 ng/mL] or hormonal therapy including oral contraceptives in the last 3 months [25,27]. We also excluded individuals with HAIRAN syndrome, defined as a fasting insulin greater than 80 μIU/mL and/or a 2 h insulin in the OGTT greater than 300 μIU/mL [28,29].

The study was approved by the Institutional Ethical Committee (code number 3 on 4 February 2025), and all the subjects gave written informed consent, in accordance with the Declaration of Helsinki.

### 2.1. Parameters Studied

Clinical and hormonal evaluation was performed between days 3 and 8 of a spontaneous menstrual cycle or after a progestin-induced withdrawal bleed [28,30], in a fasting state in the morning (8–10 a.m.) and at 2 h in a standard 75 g oral glucose tolerance test (OGTT). Weight, height and waist circumference were measured. Body mass index (BMI) was calculated as body weight (kg) divided by body height squared (m^2^). All laboratory assays were performed by commercial kits as part of the clinical protocol. Testosterone and DHEAS were measured using an automated chemiluminescence method (DXI 800- Beckman Coulter). Insulin and SHBG were measured using electrochemiluminescence immunoassay (Cobas E 601 C- Roche Diagnostics). 17α OH-progesterone was measured by using the ELISA method (DRG Diagnostics GmbH).

Insulin resistance was quantified by the homeostasis model assessment for insulin resistance, defined as HOMA-IR = [glycemia (mmol/L) × insulinemia (µU/mL)/22.5] [31]. The free-androgen (testosterone) index was calculated according to the following formula: FAI = (testosterone total (ng/mL) × 100 × 0.0347/SHBG (nmol/L)) × 100 [32].

### 2.2. Statistical Analysis

Data were analyzed using MedCalc 20.104 software (Ostend, Belgium). The results are expressed as a median (25–75th percentile) and range for continuous variables and as a number and proportion for nominal variables. We used the Kruskal–Wallis test to compare continuous variables between groups. Conover’s test was used to perform *post hoc* pairwise multiple comparisons. The nominal variables were compared by using the chi-squared test.

Correlations between continuous variables were assessed using Spearman coefficients due to non-parametric distribution of the data.

Predictors were evaluated in multivariate stepwise regressions, and the standardized beta coefficients were calculated.

BMI and age variation may affect DHEAS levels, and not all biochemical parameters evaluated as predictors were available in all PCOS cases [e.g., BMI was recorded in 240 of all 257 PCOS cases; waist in 204; total testosterone in 256; SHBG and FAI in 206; 2 h glucose and 2 h insulin were recorded together in 176 of 225 subjects who performed the OGTT]. Thus, in the aim to increase the number of subjects in a model and better understand the significant correlations, we first analyzed the models that had DHEAS as the outcome, with each predictor factor included separately in a model along with [age + BMI], and then, we studied full regression models. Similar models were applied separately in 3 population groups (all, non-obese and obese PCOS patients).

We explored the collinearity between the independent variables included in all stepwise regression models by using the variance inflation factor (VIF); values below 2 were considered acceptable, showing a moderate correlation between independent variables. A *p* value < 0.05 was considered significant.

## 3. Results

Clinical and biochemical characteristics of all PCOS cases (n = 257) are indicated in Table 1.

### 3.1. Comparative Analysis of PCOS Patients According to Their DHEAS Levels

PCOS patients were divided into tertiles according to their DHEAS levels. Comparative analysis of clinical and hormonal characteristics of the three groups is presented in Table 2.

PCOS patients with high DHEAS levels were younger and had higher 2 h insulin in the OGTT than those with low DHEAS levels [22.5 (20.25–26.75) vs. 24 (21.25–29.75) yrs, *p* < 0.05; 66.4 (38.97–114.9) vs. 45 (29.6–68.6) mU/L, *p* < 0.05, respectively], while BMI (*p* = 0.29), fasting insulin (*p* = 0.25) and HOMA-IR (*p* = 0.30) were not different between the three groups (Table 2).

### 3.2. DHEAS Correlations in PCOS

We performed univariate correlations and then multivariate stepwise regression models with the independent variables selected based on the closest correlations with DHEAS from the bivariate analysis. Body weight (BMI) and age variation may affect DHEAS levels and therefore were introduced as covariates in all regression models.

In bivariate analysis of all PCOS subjects, DHEAS correlated negatively with age (r = −0.146, *p* < 0.05) and positively with OGTT 2 h insulin level (r = 0.246, *p* < 0.001), while BMI (*p* = 0.13), fasting insulin (*p* = 0.99) and HOMA-IR (*p* = 0.87) did not correlate with DHEAS (Table 3).

When we analyzed the results according to obesity status defined based on BMI ≥ 30 kg/m^2^, the prevalence of obesity in our PCOS cases was 30.42% (73/240). DHEAS correlations were performed separately in each group of non-obese and obese PCOS, and Spearman coefficients and *p*-values are presented in detail in Table 3. The scatterplots of DHEAS bivariate analysis of non-obese and obese PCOS patients are shown comparatively in Figure S1.

We observed that DHEAS remained significantly correlated with OGTT 2 h insulin in the non-obese population with PCOS (r = 0.283, *p* < 0.01), while in obese PCOS patients, the correlation remained not significant (r = 0.097, *p* = 0.48) (Table 3 and Figure S1).

DHEAS significantly correlated with TT (r = 0.416, *p* < 0.0001) and FAI (r = 0.386, *p* < 0.0001) in all PCOS cases (Table 3) as well as in the non-obese and obese PCOS groups (Table 3 and Figure S1). FAI significantly correlated with BMI (r = 0.501, *p* < 0.0001), fasting insulin (r = 0.291, *p* < 0.0001), HOMA-IR (r = 0.298, *p* < 0.0001) and 2 h insulin in the OGTT (r = 0.373, *p* < 0.0001) in PCOS, while TT was not significantly correlated with BMI (r = 0.059, *p* = 0.36), fasting insulin (r = 0.041, *p* = 0.53), HOMA-IR (r = 0.039, *p* = 0.55) and OGTT 2 h insulin (r = 0.079, *p* = 0.29) in our patients.

### 3.3. DHEAS Multivariate Regression Analysis in PCOS

In multivariate stepwise regression analysis in all PCOS cases, 2 h insulin in the OGTT was a significant positive predictor of DHEAS (*p* = 0.0001), independent of age and BMI, and it remained significantly correlated with DHEAS in stepwise linear regression models adjusted for age, BMI, 2 h blood glucose and TT or FAI (*p* = 0.0001 and *p* = 0.009, respectively) (Table 4).

In bivariate analysis, some factors significantly correlated with DHEAS were different between the non-obese and obese populations, including 2 h insulin in the OGTT. Thus, to further explore differences between the two phenotypes, all models of DHEAS multivariate regressions evaluated in the whole population of patients were applied separately in each group of non-obese and obese PCOS patients.

In the non-obese population with PCOS, 2 h insulin in the OGTT remained a significant positive predictor of DHEAS (*p* < 0.0001), independent of age and BMI, and it was also significantly correlated with DHEAS in stepwise linear regression models adjusted for age, BMI, 2 h blood glucose and TT or FAI (*p* < 0.0001 and *p* < 0.0001, respectively) (Table 5).

In obese PCOS patients, 2 h insulin in the OGTT was not significantly correlated with DHEAS (*p* = 0.48) (Table 3 and Figure S1). Stepwise regression analysis of DHEAS in the obese population with PCOS is presented in Table 6**.**

TT and/or FAI were significant independent predictors of DHEAS in all PCOS as well as in the non-obese and obese groups of patients (Table 4, Table 5 and Table 6).

## 4. Discussion

This study indicates a positive association between stimulated insulin and DHEAS in PCOS. Two-hour insulin in the OGTT was a significant positive independent predictor of DHEAS, particularly in the non-obese population of our PCOS cohort, suggesting that hyperinsulinemia might contribute to the mechanisms causing adrenal hyperandrogenism in PCOS, and DHEAS can be a reliable marker of this modulatory influence in non-obese PCOS patients.

Although several studies reported negative or no significant associations between increased insulin and DHEAS in women with obesity and/or PCOS [12,13,20,21] and also in vitro in a human adrenocortical cell line [34], our results are consistent with data published by Amato et al. who demonstrated a significant positive correlation between DHEAS and compensatory hyperinsulinemia quantified by the oral disposition index in PCOS patients [35]. Moreover, our results are in line with previous in vivo PCOS data that suggest the effect of insulin on amplifying adrenal steroidogenesis in response to ACTH [17,18,36] and are consistent with the ability of insulin-sensitizing drugs to decrease adrenal hyperandrogenism in parallel with a decrease in insulin resistance and hyperinsulinemia in women with PCOS and in a nonhuman primate model of PCOS [37,38,39,40]. In addition, our results are in line with some [3,6] but not all [4] clinical studies that showed, by using the modern liquid chromatography–tandem mass spectrometry (LC-MS/MS) assay, that 11-oxygenated androgens, which have an adrenal source, are positively correlated with insulin and HOMA-IR [6] or obesity [3] in PCOS.

The comparative analysis of our PCOS cohort, divided into tertiles according to their DHEAS levels, was consistent with DHEAS correlations found in these patients. Specifically, we observed a significant negative association of DHEAS with age, in agreement with general data showing that adrenal steroid secretion decreases with age, including in PCOS [10,41]. In addition, PCOS patients with high DHEAS values had significantly higher serum concentrations of 2 h insulin in the OGTT than PCOS patients with low DHEAS concentrations, in line with results published by Alpañés M et al. The authors defined adrenal hyperandrogenism (AH) in PCOS by using the 95th DHEAS percentile of a control population and found that PCOS women with AH had higher circulating insulin levels and lower insulin sensitivity than their non-AH PCOS counterparts [14].

There is no consistent evidence that ovarian hormone secretion would affect adrenal steroidogenesis [10,41,42]. However, since PCOS involves common genetic factors that affect both adrenal and ovarian androgen synthesis [1], and unsurprisingly, DHEAS correlated significantly with TT and FAI in the bivariate analysis of our cases, we included these parameters in multivariate stepwise regression models, and 2 h insulin in the OGTT still remained an independent predictor of DHEAS in PCOS.

Body weight may impact adrenocortical function [10,41]. Therefore, we performed distinct correlations and regression models in the non-obese and obese populations of our PCOS and observed that 2 h insulin in the OGTT remained a significant positive predictor of DHEAS in the non-obese but not in the obese population with PCOS. Indeed, previous in vivo PCOS studies support the hypothesis that insulin would potentiate adrenal steroidogenesis [15,17] in association with a relative impairment of 17,20-lyase activity, an influence that was more evident in the 5-ene than in the 4-ene pathway [17]. In this context, we speculate that a gradual increase in the relative impairment of 17,20-lyase activity by insulin in obese populations with PCOS might limit the utility of DHEAS as a reliable marker of positive association between hyperinsulinemia and adrenal hyperandrogenism in this group of patients. Furthermore, the controversial results of previous associations between hyperinsulinemia and DHEAS in PCOS might be explained by differences in the modulatory influence of insulin on adrenal steroidogenesis as a function of prevalence of obesity in various PCOS populations, beyond the differences of age, phenotypes or the criteria used to evaluate the insulinemic secretory pattern and insulin resistance.

The pathogenic mechanisms of association between hyperinsulinemia and adrenal hyperandrogenism are not fully understood; however, previous in vitro experiments demonstrate the ability of insulin-like growth factors (insulin, IGF-I or IGF-II) to enhance the steroidogenesis and ACTH responsiveness of human adrenocortical cells in culture [43]. In addition, a mechanism involving serine phosphorylation of human P450c17 that increases adrenal androgen synthesis has been observed in vitro [44]. Considering that serine phosphorylation of the insulin receptor is one of the mechanisms of insulin resistance in PCOS [1], a unifying hypothesis was suggested for hyperandrogenism and insulin resistance, both of which are cardinal characteristics of PCOS [44]. Furthermore, it has recently been demonstrated that adrenal hyperandrogenemia but not ovarian hyperandrogenemia is mediated by insulin receptor signaling in insulin-resistant women with lipodystrophy [45]. This finding was the first demonstration in vivo of a direct role of insulin receptor signaling in regulating synthesis of adrenal androgen in humans, which may have implications for more common conditions of mild insulin resistance such as PCOS [45]. Our results are in line with these data, suggesting the stimulatory effect of insulin on PCOS-related adrenal androgen excess. While in the ovary of PCOS patients insulin most likely acts as a co-gonadotropin, enhancing the stimulating effects of LH on androgen synthesis in theca cells [46,47], increased insulin receptor signaling in adrenals should be considered for a better understanding of the controversial mechanisms of adrenal hyperandrogenism of PCOS [45], a complex genetic disease characterized by specific reproductive and metabolic abnormalities including elevated androgen levels and hyperinsulinemia which are present not only in PCOS subjects but also in their male and female relatives [1]. Indeed, stimulated levels of insulin were higher in PCOS daughters compared with control girls before the onset of ovarian [48] or adrenal hyperandrogenism [36], and elevated DHEAS values in association with markers of insulin resistance have already been demonstrated in PCOS families, including sisters [49] and brothers [50,51]. These data suggest that insulin may play an early and pivotal key role in the pathogenesis of PCOS, explaining, in part and beyond the genetics, the association between some metabolic abnormalities and underlying defects in adrenal steroidogenesis, which are heritable and are not sex specific in PCOS families [50,51,52].

We consider, in turn, some observations that DHEA and DHEAS would be partially responsible for hyperinsulinemia and insulin resistance. For instance, it has been shown in vitro in the MIN6 cell that DHEAS may exert direct stimulatory effects on insulin secretion partly via AMP-activated protein kinase inhibition and Acetyl CoA carboxylase-1 upregulation [53]. However, there is no data to demonstrate such a mechanism in humans. On the other hand, DHEA increased insulin binding to its receptor in vitro [54], and in vivo, DHEAS correlated negatively with insulin resistance in females with obesity and type 2 diabetes mellitus but not in obese females without type 2 diabetes mellitus [55]. In addition, low DHEAS was associated with coronary artery disease [56] and progression to type 2 diabetes mellitus [57] in men. Considering these data, a negative association between DHEAS and insulin resistance would be expected in PCOS, contrary to the results of our study. Overall, the mechanisms by which DHEAS would affect insulin resistance in PCOS patients are unknown.

This study has limitations, which include the following: (1) The evaluation of insulin resistance/hyperinsulinemia was performed by measurement of glucose and insulin in a fasting state and at 2 h in a standard OGTT; therefore, we have limits in identifying correlations for insulin resistance/hyperinsulinemia in PCOS. (2) The method for assessing total testosterone was not LC-MS/MS assay, which is the method currently recommended [58]. (3) Although the prevalence of obesity in this study is consistent with other PCOS populations [59], the significance of DHEAS analysis in obese PCOS patients, including the association between insulin and their DHEAS levels, may be hampered by the low number of individuals in the obese PCOS group. In statistical terms, our observations need additional validation by future studies with a higher number of obese PCOS patients. (4) The cross-sectional design does not include interventions to test the causal mechanisms underlying the correlations that were observed.

In conclusion, our data evidence a significant positive association of stimulated insulin with DHEAS in PCOS, particularly in the non-obese population of PCOS. Whether this relationship is the result of a direct effect of insulin action at the level of synthesis of adrenal androgens, or whether both elevated DHEAS as marker of adrenal hyperandrogenism and hyperinsulinemia are driven by a common still undetermined mechanism, remains to be thoroughly investigated and determined in the near future.

## Figures and Tables

**Table 1:** Clinical and biochemical characteristics of PCOS cases (n = 257).

Characteristics	Median (25–75th Percentile)	Observed Range
Age (yrs)	23 (21–27)	16–41
BMI (kg/m^2^)	24.78 (20.83–31.23)	15.23–48.11
Obesity (BMI ≥ 30 kg/m^2^) ^#^	73/240 (30.42%)	-
Waist (cm)	83 (73.6–100)	60–126
mFG score	7 (3–10)	0–34
LH (U/L)	8.88 (5.31–13.13)	1.46–49.4
FSH (U/L)	6.24 (4.83–7.54)	0.7–15.04
E2 (pg/mL)	48.5 (35.2–68)	2.46–413
Total testosterone (ng/mL)	0.72 (0.55–0.88)	0.09–2.56
SHBG (nmol/L)	42.73 (26.37–69.58)	4.5–278.05
FAI	5.83 (3.35–9.75)	0.55–57.2
DHEAS (µg/dL)	267.9 (185.5–353.35)	20.04–810.9
17OH progesterone (ng/mL)	1.03 (0.76–1.31)	0.29–1.94
Fasting glucose (mg/dL)	84.4 (79.25–91)	45–133
Fasting insulin (mU/L)	14.4 (8.75–23.4)	1–69
HOMA-IR	2.98 (1.84–5.11)	0.19–15.53
2 h glucose (mg/dL)	99 (86.1–114)	39–203.5
2 h insulin (mU/L)	54 (31.7–98)	4–252
SBP (mmHg)	110 (100–120)	85–180
DBP (mmHg)	70 (60–80)	50–150
Triglycerides (mg/dL)	79.25 (58–120.5)	29–470
HDL cholesterol (mg/dL)	52 (43–62.75)	16–110
MetS ^#^	51/237 (21.52%)	-

Data are shown as median (25–75th percentile) and observed range. ^#^ Data are shown as number (proportion). BMI, body mass index; mFG score, modified Ferriman–Gallwey score; LH, luteinizing hormone; FSH, follicle-stimulating hormone; E2, estradiol; SHBG, sex hormone-binding globulin; FAI, free-androgen index; DHEAS, dehydroepiandrosterone sulfate; 2 h glucose and 2 h insulin, glucose and insulin at 2 h during standard oral glucose tolerance test (OGTT); HOMA-IR, homeostasis model assessment for insulin resistance; SBP and DBP, systolic and diastolic blood pressure; MetS, metabolic syndrome according to the IDF 2009 harmonized criteria [33].

**Table 2:** Clinical and biochemical characteristics of PCOS patients according to their DHEAS levels.

	Lower Tertile DHEAS ≤ 216.7 (n = 86)	Middle Tertile DHEAS = 216.8–317.2 (n = 86)	Upper Tertile DHEAS ≥ 318.8 (n = 85)	*p*
DHEAS (µg/dL)	152.85 (123.92–186.72) ^a^	268.8 (244.45–293.75) ^b^	397.9 (353.35–479.8) ^c^	<0.001
Age (yrs)	24 (21.25–29.75) ^a^	23 (21–27) ^a,b^	22.5 (20.25–26.75) ^b^	<0.05
BMI (kg/m^2^)	24.05 (20.25–31.87)	23.98 (20.96–29.81)	26.21 (21.7–32)	0.29
Waist (cm)	83 (71–94)	80 (73.27–95.5)	88.5 (75.62–103.75)	0.16
Total testosterone (ng/mL)	0.63 (0.44–74) ^a^	0.72 (0.56–0.83) ^b^	0.83 (0.7–1.03) ^c^	<0.001
SHBG (nmol/L)	54.3 (34.9–81.09) ^a^	41.38 (25.87–59.92) ^a,b^	35.3 (19.67–56.95) ^b^	<0.01
FAI	4.24 (2.13–7.27) ^a^	5.16 (3.51–9.82) ^b^	8.1 (4.46–15.5) ^c^	<0.001
Fasting glucose (mg/dL)	84.1 (78.25–90.15)	85.25 (80.45–91)	83.4 (79.25–92.3)	0.59
Fasting insulin (mU/L)	16 (9.9–23)	13 (7.08–23.4)	15 (9.81–26.11)	0.25
HOMA-IR	3.11 (2.13–4.98)	2.66 (1.48–4.97)	3.26 (2.07–5.88)	0.30
2 h glucose (mg/dL)	92.25 (79.55–105) ^a^	99.8 (88.37–118) ^b^	103.7 (91.75–123.55) ^b^	0.001
2 h insulin (mU/L)	45 (29.6–68.6) ^a^	54.95 (32–117.5) ^a,b^	66.4 (38.97–114.9) ^b^	<0.05

Data are shown as median (25–75th percentile). *p* values indicate differences between groups as analyzed by Kruskal–Wallis test; ^a–c^ Significant differences between any two groups (*p* < 0.05), as demonstrated by Conover’s *post hoc* test, are indicated with different superscript letters. DHEAS, dehydroepiandrosterone sulfate; BMI, body mass index; SHBG, sex hormone-binding globulin; FAI, free-androgen index; HOMA-IR, homeostasis model assessment for insulin resistance.

**Table 3:** Univariate Spearman correlations of DHEAS in PCOS.

	All PCOS (n = 257)		Non-Obese PCOS (n = 167)		Obese PCOS (n = 73)	
Variables	r	*p*	r	*p*	r	*p*
Age (yrs)	−0.146	<0.05	−0.073	0.35	−0.317	<0.01
BMI (kg/m^2^)	0.098	0.13	0.147	0.07	−0.031	0.79
Waist (cm)	0.105	0.14	0.044	0.61	0.037	0.77
Fasting glucose (mg/dL)	0.021	0.74	0.028	0.72	−0.067	0.57
Fasting insulin (mU/L)	−0.0001	0.99	−0.091	0.27	−0.009	0.94
HOMA-IR	0.0105	0.87	−0.072	0.38	−0.004	0.97
2 h glucose (mg/dL)	0.231	<0.001	0.334	<0.0001	0.006	0.96
2 h insulin (mU/L)	0.246	<0.001	0.283	<0.01	0.097	0.48
Total testosterone (ng/mL)	0.416	<0.0001	0.473	<0.0001	0.341	<0.01
FAI	0.386	<0.0001	0.387	<0.0001	0.504	<0.0001

Spearman coefficients of correlation and their *p*-values are presented. DHEAS, dehydroepiandrosterone sulfate; BMI, body mass index; HOMA-IR, homeostasis model assessment for insulin resistance; FAI, free-androgen index.

**Table 4:** Predictors of DHEAS, multivariate stepwise regression analysis in all PCOS cases (n = 257).

	Model 1 (n = 171)	Model 2 (n = 212)	Model 3 (n = 236)	Model 4 (n = 187)	Model 5 (n = 165)	Model 6 (n = 130)
Age	−0.148 *p* = 0.0439	−0.160 *p* = 0.018	−0.159 *p* = 0.009	−0.239 *p* = 0.0004	−0.160 *p* = 0.0261	−0.197 *p* = 0.014
BMI	- *p* = NS	- *p* = NS	- *p* = NS	- *p* = NS	- *p* = NS	- *p* = NS
2 h insulin	0.286 *p* = 0.0001	X	X	X	0.280 *p* = 0.0001	0.214 *p* = 0.009
2 h glucose	X	0.247 *p* = 0.0003	X	X	- *p* = NS	- *p* = NS
Total testosterone	X	X	0.321 *p* < 0.0001	X	0.245 *p* = 0.0009	X
FAI	X	X	X	−0.398 *p* < 0.0001	X	0.331 *p* = 0.0001
R^2^ (adjusted)	0.10 (*p* < 0.0001)	0.067 (*p* = 0.0002)	0.12 (*p* < 0.0001)	0.192 (*p* < 0.0001)	0.15 (*p* < 0.0001)	0.20 (*p* < 0.0001)

Age and BMI were introduced as covariates in all regression models. In addition, 2 h insulin, 2 h glucose and Total testosterone or FAI were the additional independent variables included in regression analyses, as shown in the models above. Data represent standardized beta coefficients, adjusted R^2^ values and significant *p*-values; the non-significant *p* values were noted as NS.

**Table 5:** Predictors of DHEAS, multivariate stepwise regression analysis in non-obese PCOS cases (n = 167).

	Model 1 (n = 116)	Model 2 (n = 143)	Model 3 (n = 163)	Model 4 (n = 131)	Model 5 (n = 112)	Model 6 (n = 89)
Age	- *p* = NS	- *p* = NS	- *p* = NS	−0.174 *p* = 0.039	- *p* = NS	- *p* = NS
BMI	- *p* = NS	- *p* = NS	- *p* = NS	- *p* = NS	- *p* = NS	- *p* = NS
2 h insulin	0.370 *p* < 0.0001	X	X	X	0.407 *p* < 0.0001	0.438 *p* < 0.0001
2 h glucose	X	0.323 *p* = 0.0001	X	X	- *p* = NS	- *p* = NS
Total testosterone	X	X	0.311 *p* = 0.0001	X	0.199 *p* = 0.022	X
FAI	X	X	X	0.311 *p* = 0.0003	X	- *p* = NS
R^2^ (adjusted)	0.13 (*p* < 0.0001)	0.098 (*p* = 0.0001)	0.092 (*p* = 0.0001)	0.10 (*p* = 0.0004)	0.18 (*p* < 0.0001)	0.18 (*p* < 0.0001)

Age and BMI were introduced as covariates in all regression models. In addition, 2 h insulin, 2 h glucose and Total testosterone or FAI were the additional independent variables included in regression analyses, as shown in the models above. Data represent standardized beta coefficients, adjusted R^2^ values and significant *p*-values; the non-significant *p* values were noted as NS.

**Table 6:** Predictors of DHEAS, multivariate stepwise regression analysis in obese PCOS (n = 73).

	Model 1 (n = 55)	Model 2 (n = 69)	Model 3 (n = 73)	Model 4 (n = 56)	Model 5 (n = 53)	Model 6 (n = 41)
Age	−0.293 *p* = 0.029	−0.326 *p* = 0.006	−0.281 *p* = 0.01	−0.311 *p* = 0.007	−0.262 *p* = 0.039	−0.358 *p* = 0.01
BMI	- *p* = NS	- *p* = NS	- *p* = NS	- *p* = NS	- *p* = NS	- *p* = NS
2 h insulin	- *p* = NS	X	X	X	- *p* = NS	- *p* = NS
2 h glucose	X	- *p* = NS	X	X	- *p* = NS	- *p* = NS
Total testosterone	X	X	0.328 *p* = 0.002	X	0.383 *p* = 0.003	X
FAI	X	X	X	0.452 *p* = 0.0002	X	0.403 *p* = 0.004
R^2^ (adjusted)	0.068 (*p* = 0.029)	0.092 (*p* = 0.006)	0.18 (*p* = 0.0003)	0.31 (*p* = 0.0001)	0.20 (*p* = 0.001)	0.27 (*p* = 0.0008)

Age and BMI were introduced as covariates in all regression models. In addition, 2 h insulin, 2 h glucose and Total testosterone or FAI were the additional independent variables included in regression analyses, as shown in the models above. Data represent standardized beta coefficients, adjusted R^2^ values and significant *p*-values; the non-significant *p* values were noted as NS.

## Data Availability

The data presented in this study are available on request from the corresponding author (the data are not publicly available due to privacy and ethical restrictions).

## Supplementary Materials

The following supporting information can be downloaded at: https://www.mdpi.com/article/10.3390/jcm14176246/s1, Figure S1: The scatterplots of DHEAS bivariate analysis of non-obese and obese PCOS patients.
