# Supplementary material for: Stimulated Hyperinsulinemia Is Independently Associated with Higher Serum DHEAS in PCOS: A Retrospective Study
Source: J Clin Med. 2025 Sep 4;14(17):6246. doi: 10.3390/jcm14176246 (PMC12429209; doi:10.3390/jcm14176246)
Supplement: Supplementary file 1 [file jcm-14-06246-s001.zip › jcm-3784262-supplementary.pdf]

Figure S1.

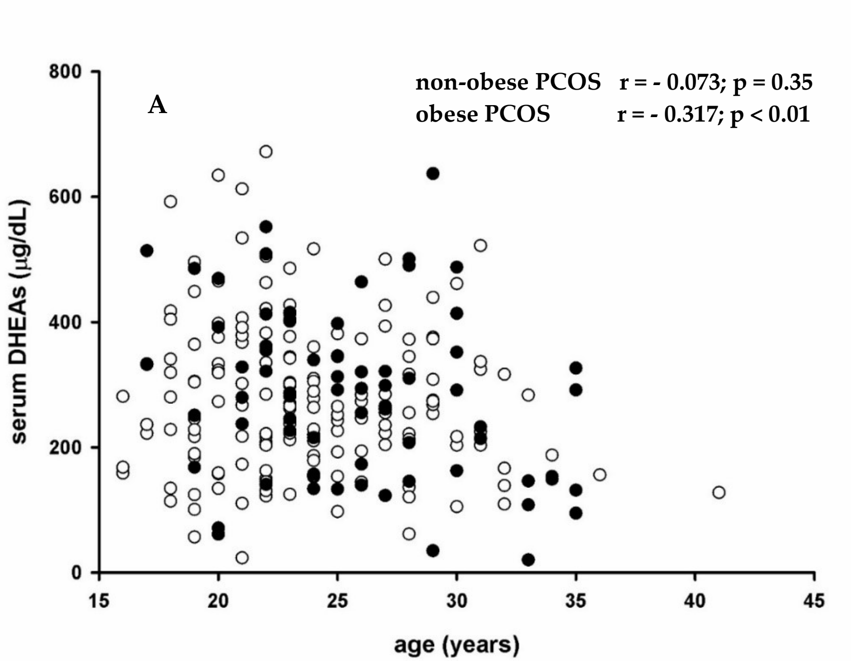

(A) Scatterplot of DHEAS *versus* age in non-obese (empty circles) and obese (black circles) PCOS. Spearman r- and p-values are shown on the graph.

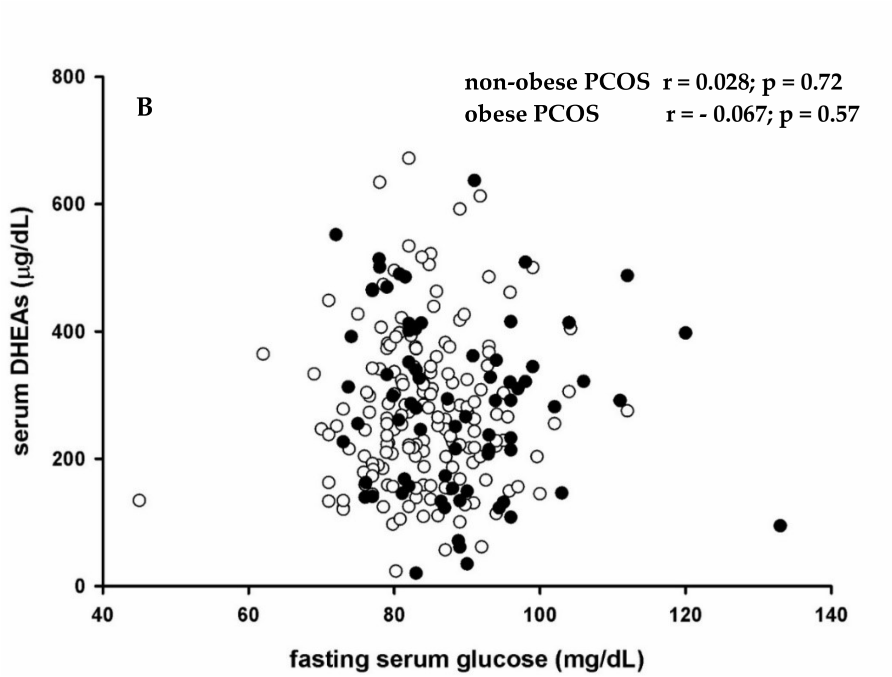

(B) Scatterplot of DHEAS *versus* fasting glucose in non-obese (empty circles) and obese (black circles) PCOS. Spearman r- and p-values are shown on the graph.

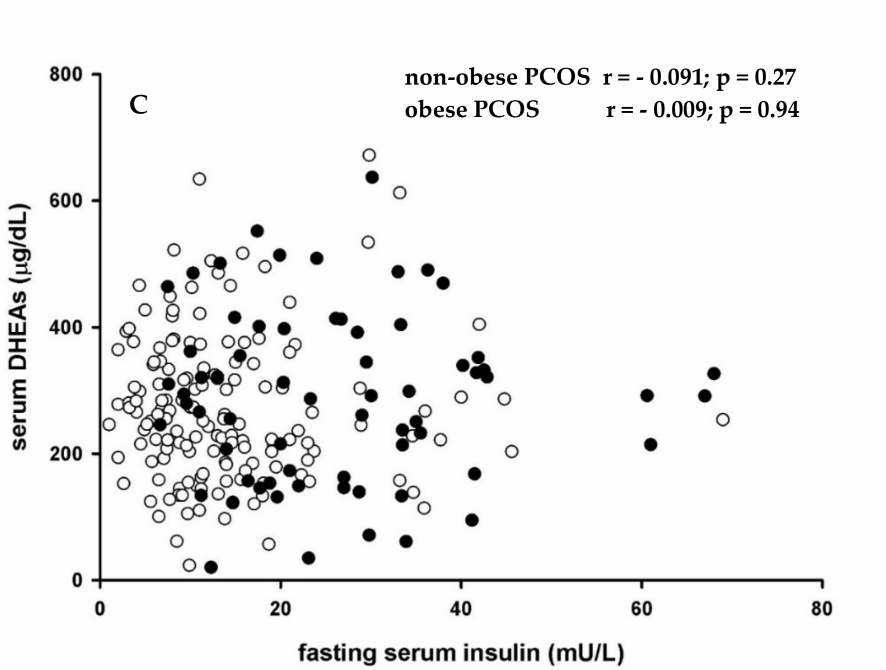

(C) Scatterplot of DHEAS *versus* fasting insulin in non-obese (empty circles) and obese (black circles) PCOS. Spearman r- and p-values are shown on the graph.

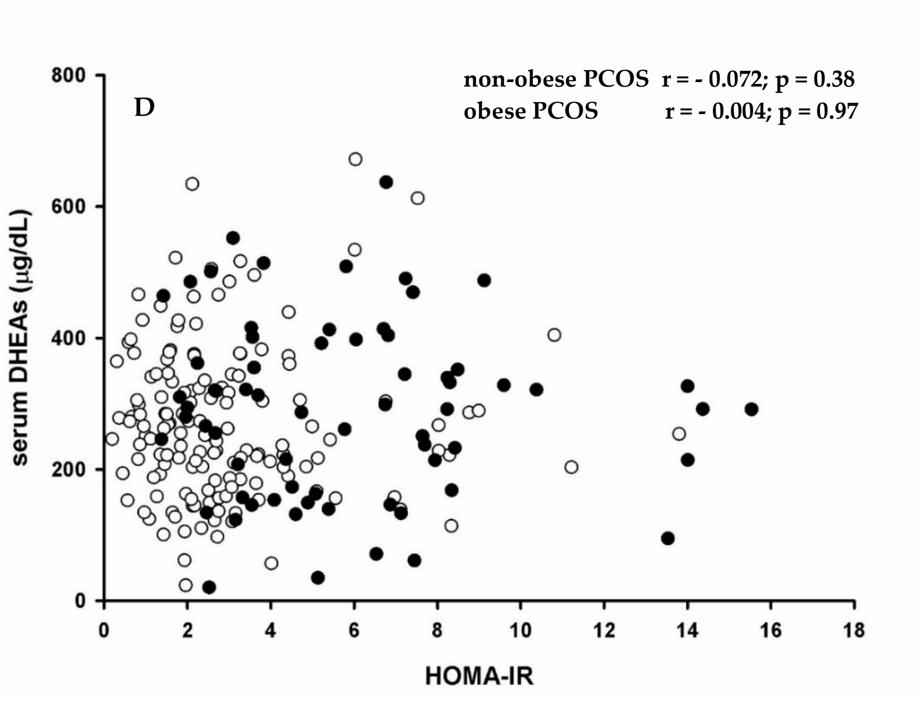

(D) Scatterplot of DHEAS *versus* HOMA-IR in non-obese (empty circles) and obese (black circles) PCOS. Spearman r- and p-values are shown on the graph.

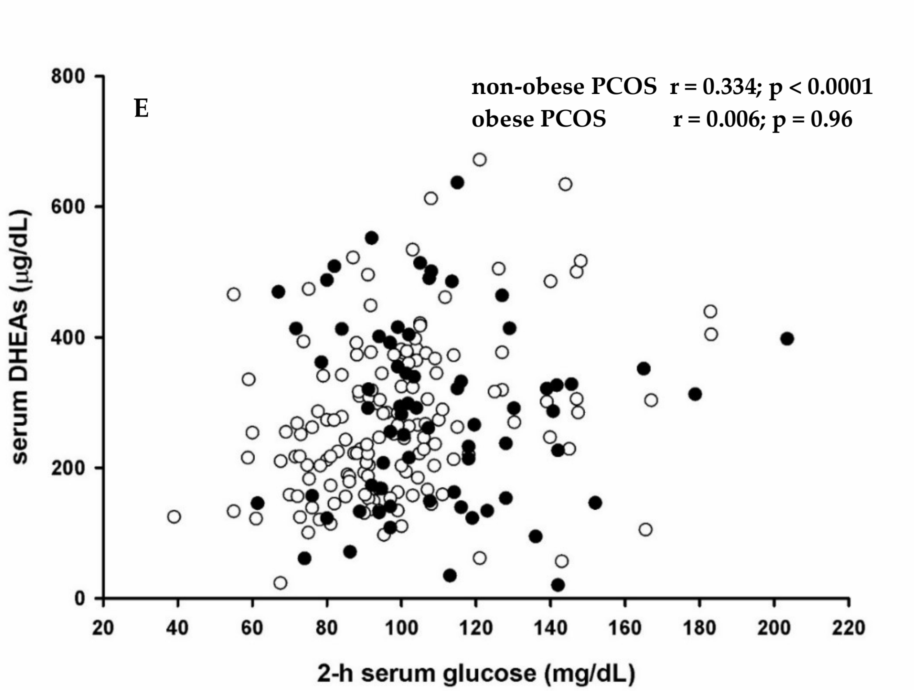

(E) Scatterplot of DHEAS *versus* OGTT 2-h glucose in non-obese (empty circles) and obese (black circles) PCOS. Spearman r- and p-values are shown on the graph.

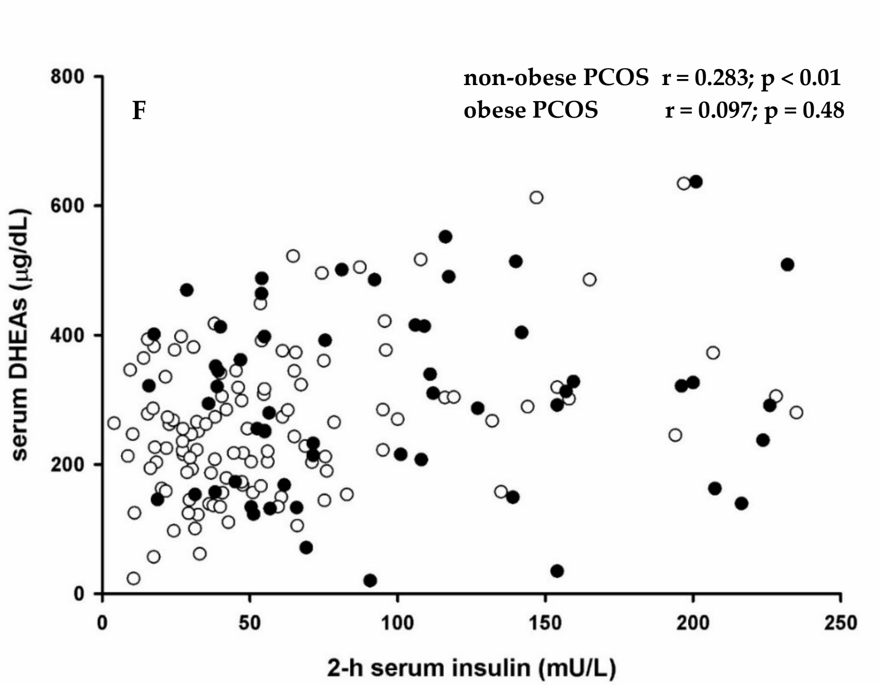

(F) Scatterplot of DHEAS *versus* OGTT 2-h insulin in non-obese (empty circles) and obese (black circles) PCOS. Spearman r- and p-values are shown on the graph.

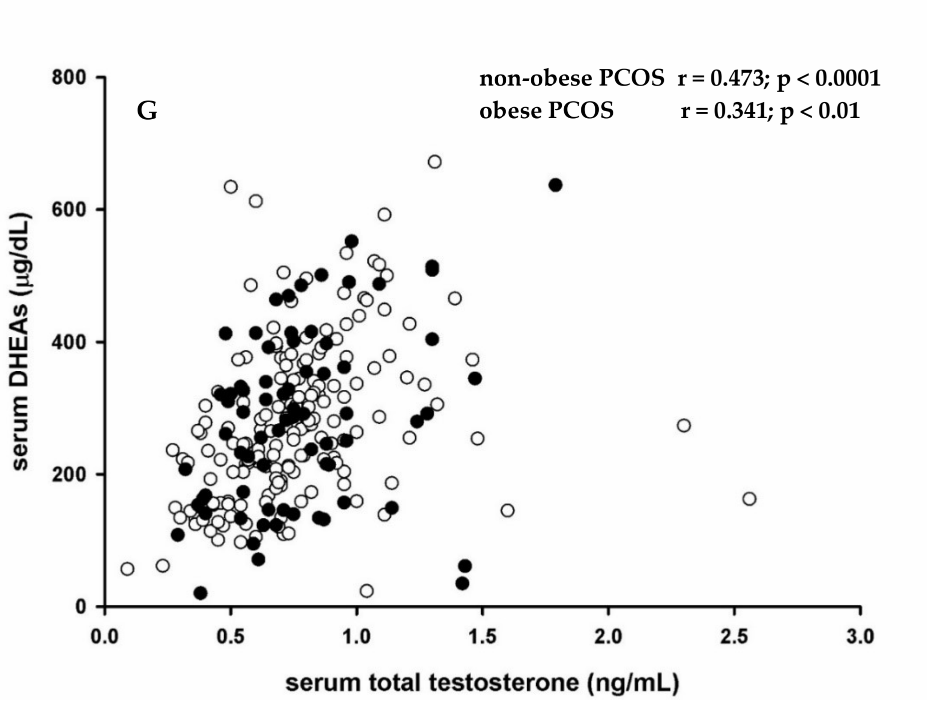

(G) Scatterplot of DHEAS *versus* total testosterone in non-obese (empty circles) and obese (black circles) PCOS. Spearman r- and p-values are shown on the graph.

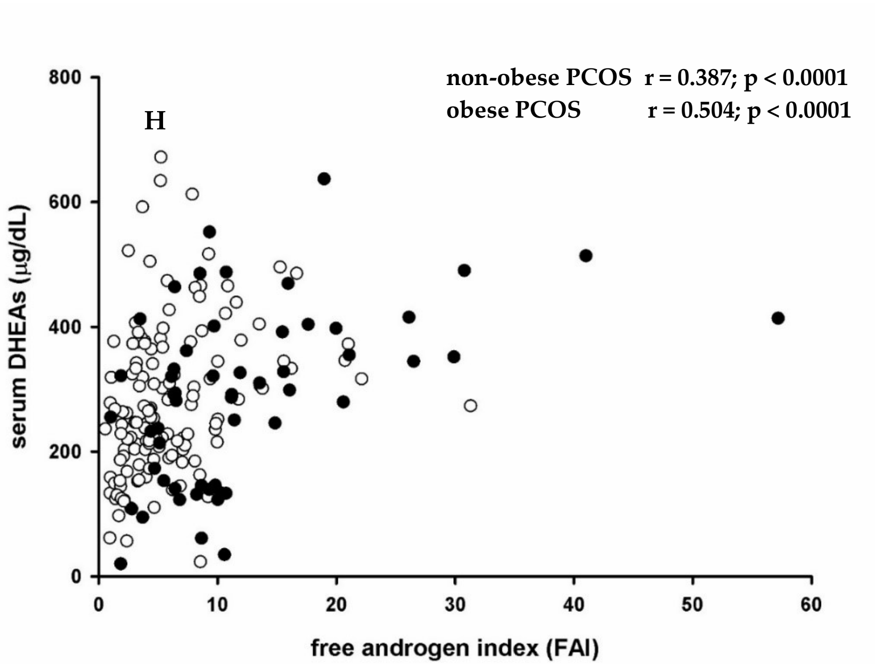

(H) Scatterplot of DHEAS *versus* free androgen index (FAI) in non-obese (empty circles) and obese (black circles) PCOS. Spearman r- and p-values are shown on the graph.
